# A Barrierless Pathway Accessing the C_9_H_9_ and C_9_H_8_ Potential Energy Surfaces via the Elementary Reaction of Benzene with 1-Propynyl

**DOI:** 10.1038/s41598-019-53987-5

**Published:** 2019-11-26

**Authors:** Aaron M. Thomas, Srinivas Doddipatla, Ralf I. Kaiser, Galiya R. Galimova, Alexander M. Mebel

**Affiliations:** 1 Department of Chemistry, University of Hawai’i at Manoa, Honolulu, Hawaii 96822 United States; 2 Samara National Research University, Samara, 443086 Russia; 3 Department of Chemistry and Biochemistry, Florida International University, Miami, Florida 33199 United States

**Keywords:** Laboratory astrophysics, Chemical physics, Reaction kinetics and dynamics, Reaction mechanisms

## Abstract

The crossed molecular beams reactions of the 1-propynyl radical (CH_3_CC; X^2^A_1_) with benzene (C_6_H_6_; X^1^A_1g_) and D6-benzene (C_6_D_6_; X^1^A_1g_) were conducted to explore the formation of C_9_H_8_ isomers under single-collision conditions. The underlying reaction mechanisms were unravelled through the combination of the experimental data with electronic structure and statistical RRKM calculations. These data suggest the formation of 1-phenyl-1-propyne (C_6_H_5_CCCH_3_) via the barrierless addition of 1-propynyl to benzene forming a low-lying doublet C_9_H_9_ intermediate that dissociates by hydrogen atom emission via a tight transition state. In accordance with our experiments, RRKM calculations predict that the thermodynamically most stable isomer – the polycyclic aromatic hydrocarbon (PAH) indene – is not formed via this reaction. With all barriers lying below the energy of the reactants, this reaction is viable in the cold interstellar medium where several methyl-substituted molecules have been detected. Its underlying mechanism therefore advances our understanding of how methyl-substituted hydrocarbons can be formed under extreme conditions such as those found in the molecular cloud TMC-1. Implications for the chemistry of the 1-propynyl radical in astrophysical environments are also discussed.

## Introduction

**Figure 1:**
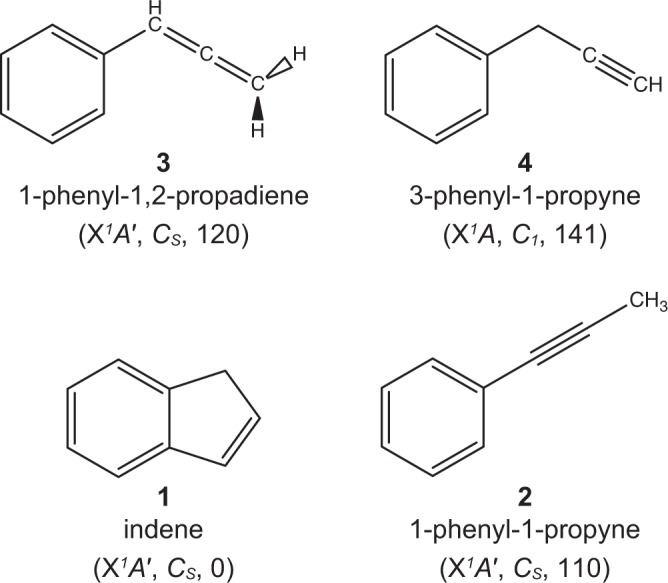
Energetically low-lying structural isomers of the C_9_H_8_ molecule. Enthalpies of formation (∆_f_*H*(0 K)) are given in kJ mol^−1^ relative to indene.

**Figure 2:**
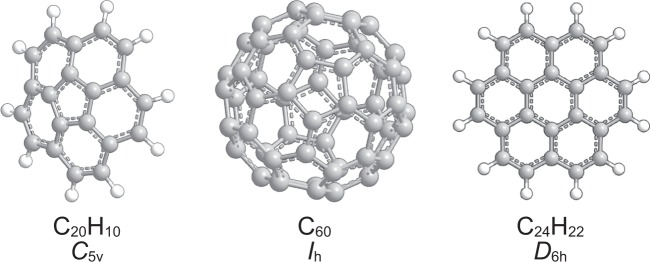
Corannulene (C_20_H_10_), Buckminsterfullerene (C_60_), and Coronene (C_24_H_12_).

During the last decades, the C_9_H_9_ and C_9_H_8_ potential energy surfaces (PES) have received considerable attention from the astrochemistry, combustion, and chemical reaction dynamics communities in exploring the formation of indene (C_9_H_8_; [1]) along with its 1-phenyl-1-propyne (C_6_H_5_CCCH_3_; [2]), 1-phenyl-1,2-propadiene (C_6_H_5_CHCCH_2_; [3]), and 3-phenyl-1-propyne (C_6_H_5_CH_2_CCH; [4]) isomers in the interstellar medium (ISM) and in combustion systems (Fig. 1)^1–11^. Indene [1] represents the simplest prototype of a polycyclic aromatic hydrocarbon (PAH) containing a partially saturated pentagon fused with a benzene ring^12–14^. The carbon backbone of the indene molecule as found in corannulene (C_20_H_10_) and fullerenes like Buckminsterfullerene (C_60_) is central to the transition of planar PAHs like coronene (C_24_H_12_) (Fig. 2) to three-dimensional carbonaceous nanostructures and eventually soot in combustion systems and in deep space^15–18^. Therefore, the elementary reactions underlying the initial synthesis of PAHs carrying five-membered ring(s) is central to a complete understanding of how three-dimensional (bowl-shaped) nanostructures and ultimately soot particles are formed in extreme environments such as combustion systems and the ISM.

Recent molecular beam experiments along with electronic structure calculations have been influential in exploring these early stages of chemistry via the C_9_H_9_ and C_9_H_8_ potential energy surfaces (PES). Crossed molecular beams experiments within the phenyl (C_6_H_5_)–methylacetylene (CH_3_CCH) system revealed the formation of 1-phenyl-1-propyne [2] at collision energies between 91–161 kJ mol^−1^ via short lived doublet C_9_H_9_ reaction intermediates^8–11^. Crossed beams studies of the phenyl (C_6_H_5_)–allene (H_2_CCCH_2_) system exposed similar dynamics via phenyl addition to a terminal carbon atom followed by hydrogen atom elimination through a tight exit transition state and 1-phenyl-1,2-propadiene [3] formation^10,11^. These elementary reactions must overcome entrance barriers between 6 and 26 kJ mol^−1^ ^10,11^ and therefore can only operate at elevated temperatures of combustion flames and in circumstellar envelopes close to the central carbon star where temperatures up to 2,500 K reside, but not in cold molecular clouds (10 K)^19^. Complementary high temperature chemical microreactor studies at 600 K exploiting vacuum ultraviolet (VUV) light to photoionize the reaction products reveal that the benzyl radical (C_6_H_5_CH_2_) was found to react with acetylene (C_2_H_2_) yielding solely indene [1]^6^. The inherent entrance barrier to reaction of 51 kJ mol^−1^ is much higher than the barriers in the phenyl - allene and phenyl - methylacetylene systems since the benzyl radical is resonance stabilized, but the phenyl radical is not; this leads to the loss of the resonance stabilization energy of 51 kJ mol^−1^ upon addition of the benzyl radical to the acetylene molecule^6^. Contemporary crossed molecular beams experiments at lower collision energies of about 45 kJ mol^−1^ and high temperature chemical reactor studies at 1,200–1,500 K along with sophisticated isotopic substitution experiments provided compelling evidence that the phenyl radical reacts with allene (C_3_H_4_) and methylacetylene (C_3_H_4_) forming indene (C_9_H_8_) [1] along with non-PAH isomers 1-phenyl-1-propyne [2], 1-phenyl-1,2-propadiene [3], and possibly 3-phenyl-1-propyne [4]^1,5^. Generally, these studies exposed that elementary reactions of acetylene and allene/methylacetylene with aromatic radicals can access the C_9_H_9_ and C_9_H_8_ PESs. However, although the overall reactions were exoergic between 7 and 148 kJ mol^−1^, these studies suggest that the synthesis of *any* C_9_H_8_ isomer involves an entrance barrier to addition between 6 and 51 kJ mol^−1^ thus blocking these pathways in cold molecular clouds like Taurus (TMC-1) and Orion (OMC-1)^20–22^.

Here, we present the results of crossed molecular beams experiments of the 1-propynyl radical (CH_3_CC; X^2^A_1_) with benzene (C_6_H_6_; X^1^A_1g_) and D6-benzene (C_6_D_6_; X^1^A_1g_) and combine these data with novel electronic structure calculations on the C_9_H_9_ PES. These studies reveal an overall exoergic, but in contrast to previous systems, *entrance-barrierless* reaction accessing the C_9_H_9_ surface via addition of the 1-propynyl radical to the benzene ring leading eventually to the formation of the 1-phenyl-1-propyne product (C_6_H_5_CCCH_3_) along with atomic hydrogen. Our investigations propose that 1-phenyl-1-propyne could exist and be readily observable in cold molecular clouds. Methylacetylene, which has been observed toward TMC-1^23^, is known to fragment to 1-propynyl upon interaction with ultraviolet radiation^24–26^. If formed, the 1-propynyl radical could react rapidly with benzene^27^ upon collision to yield 1-phenyl-1-propyne which may be susceptible to observation due to its large dipole moment of 0.48 Debye.

## Experimental Results

### Laboratory frame

**Figure 3:**
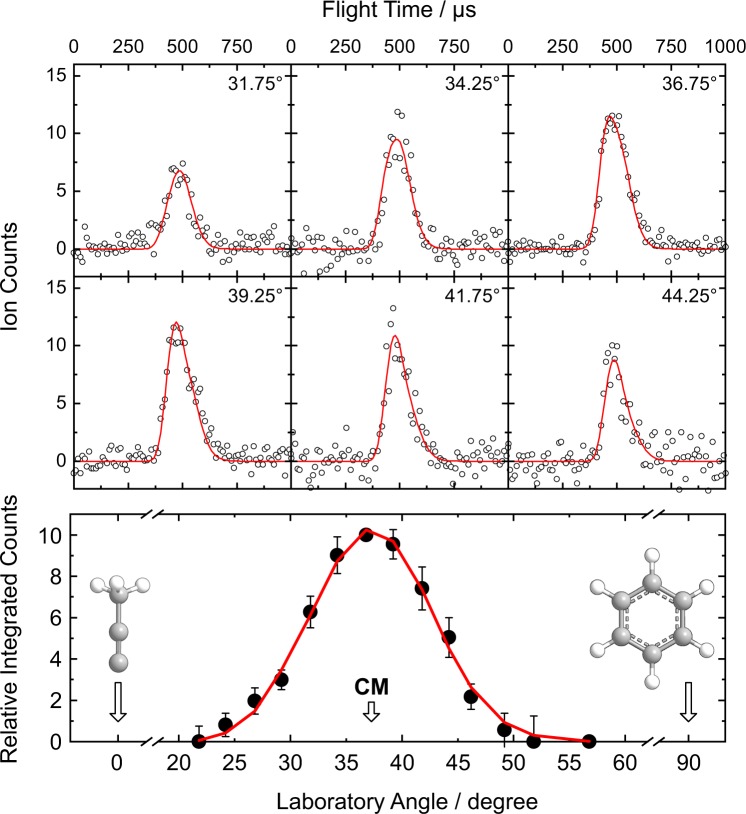
(**a**) Time-of-flight spectra and (**b**) laboratory angular distribution recorded at *m/z* 116 (C_9_H_8_^+^) for products formed in the reaction of the 1-propynyl radical with benzene. The circles represent the experimental data and the solid lines the best fits.

We monitored potential products formed from the reactive scattering of the 1-propynyl radical (CH_3_CC; 39 amu) with benzene (C_6_H_6_; 78 amu) along Θ_CM_ at *m/z* = 117 (C_9_H_9_^+^) and 116 (C_9_H_8_^+^) to assess the formation of a persistent reaction intermediate (adduct) and/or hydrogen loss reaction product, respectively. Signals were observed for each *m/z* value. However, the TOF spectra recorded at each *m/z* depict an identical pattern after scaling and thus originate from the same channel, namely the formation of C_9_H_8_ (116 amu) by elimination of atomic hydrogen (1 amu). The signal at *m/z* = 117 therefore arises from the natural distribution of carbon atom isotopes yielding ^13^CC_8_H_8_ occurring at level of about 9.9%. Following confirmation of the atomic hydrogen loss product channel, we recorded 5.6 × 10^6^ TOF spectra of nascent C_9_H_8_ in the LAB frame at *m/z* = 116 in 2.5° intervals from 21.75° to 58.25° (Fig. 3). The TOF spectra were then normalized with respect to Θ_CM_ and integrated to yield the laboratory angular distribution (Fig. 3), which is nearly symmetric around Θ_CM_ thereby suggesting the presence of indirect reaction dynamics via one or more relatively long-lived C_9_H_9_ intermediate(s) preceding dissociation to C_9_H_8_ plus atomic hydrogen. The laboratory data were fit by forward convolution using a single reaction channel, namely CH_3_CC (39 amu) + C_6_H_6_ (78 amu) → C_9_H_8_ (116 amu) + H (1 amu) with a reaction cross section proportional to *E*_C_^−1/3^ for a barrier-less reaction within the line-of-centre model (section 5)^28^.

**Figure 4:**
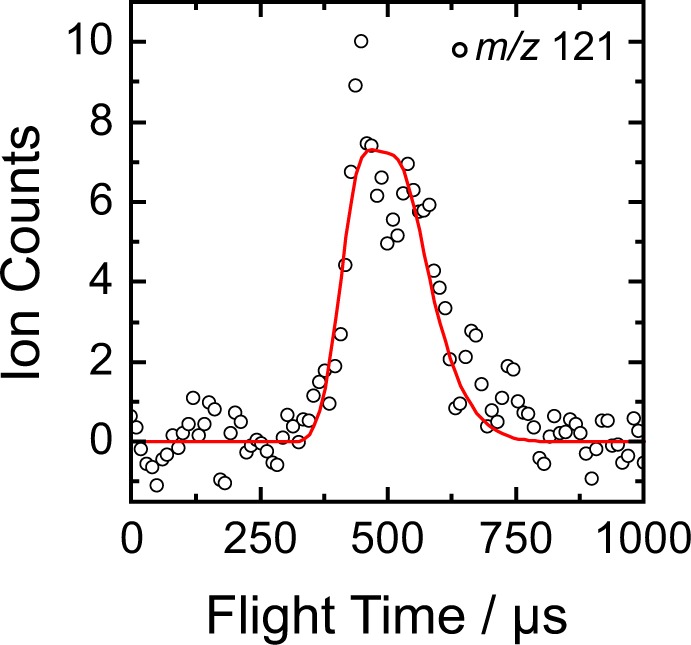
Time-of-flight (TOF) spectra for the reaction of the 1-propynyl radical with D6-benzene at *m/z* = 121 (C_9_H_3_D_5_^+^). The open circles represent the experimental data, and the red line represents the fit obtained from the forward-convolution routine.

To trace the source of the hydrogen atom emission, we substituted the benzene reactant with D6-benzene (C_6_D_6_) and performed a second experiment with CH_3_CC (39 amu) plus C_6_D_6_ (84 amu) to determine if the atom is lost from the 1-propynyl or benzene reactant. We probed the atomic hydrogen and atomic deuterium loss channels along Θ_CM_ at *m/z* = 122 (C_9_H_2_D_6_^+^) and 121 (C_9_H_3_D_5_^+^), respectively. A very strong signal was recorded at *m/z* 121 while a weaker signal was discernible at *m/z* = 122. By comparison with the ratios recovered in the CH_3_CC - C_6_H_6_ reaction for the ^13^CC_8_H_8_/C_9_H_8_ product(s), we conclude that the TOF spectrum at *m/z* = 122 owes to ^13^C-enrichment in the form of ^13^CC_8_H_3_D_5_ and that only the atomic deuterium loss channel to form C_9_H_3_D_5_ is observed. Importantly, the TOF recorded at Θ_CM_ can be fit using the CM functions derived from the hydrogenated system, which suggests the products formed in each experiment are isotopologues (Fig. 4).

### Centre-of-mass frame

**Figure 5:**
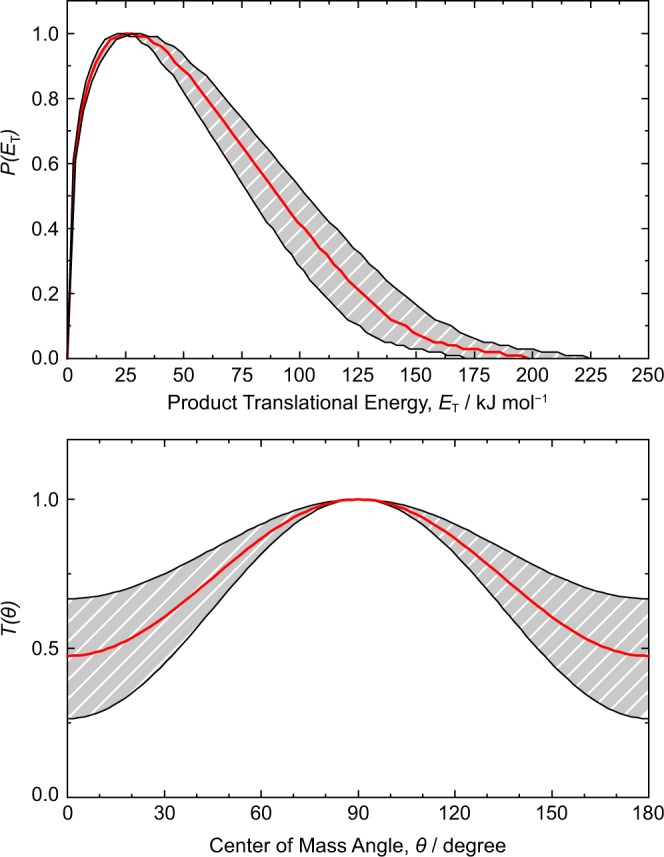
The *(top)* center-of-mass translational energy *P*(*E*_T_) and *(bottom)* angular *T(θ)* flux distributions for the reaction of the 1-propynyl radical with benzene forming C_9_H_8_ isomer(s) by atomic hydrogen emission. Solid red lines represent the best fits while shaded areas indicate the experimental error limits.

In the laboratory frame, it is clear that the reaction product(s) are formed via the loss of an aromatic hydrogen atom forming one or more C_9_H_8_ isomers. Details of the reaction coordinate are encoded in the product distribution and can be revealed in the centre-of-mass (CM) frame, from which we now examine via the CM translational energy *P(E*_T_) and angular *T(θ*) flux distributions (Fig. 5). The *P(E*_T_) terminates at 198 ± 27 kJ mol^−1^ and represents the maximum energy available *E*_avail_ to the reaction system. Taken together with the collision energy *E*_C_ of 40.8 ± 0.5 kJ mol^−1^, we obtain a reaction energy for this process of ∆_r_*G* = −157 ± 27 kJ mol^−1^, where these quantities have been related through energy conservation via *E*_avail_ = *E*_C_ − ∆_r_*G*. While the most probable *E*_T_ occurs near 28 ± 4 kJ mol^−1^, nascent C_9_H_8_ products carry an average translational energy of 57 ± 8 kJ mol^−1^, suggesting that only about 29 ± 6% of *E*_avail_ is disposed into translational degrees of freedom. The high fraction of energy appearing as rovibrational excitation and nonzero peaking of the *P(E*_T_) are markers for a reaction mechanism that forms products indirectly via activated C_9_H_9_ intermediate(s) that must overcome a barrier to disscoiation^28^. The symmetry of the *T(θ*) distribution about *θ* = 90° along with the nonzero intensity at all angles together indicate the reaction proceeds through a long-lived C_9_H_9_ intermediate complex. Furthermore, the maximum in the distribution at *θ* = 90° strongly suggests the C_9_H_9_ intermediate decomposes via a transition state that emits atomic hydrogen perpendicular to its rotational plane, i.e. parallel to the total angular momentum vector as defined by the initial conditions of the experiment^28,29^.

## Discussion

**Figure 6:**
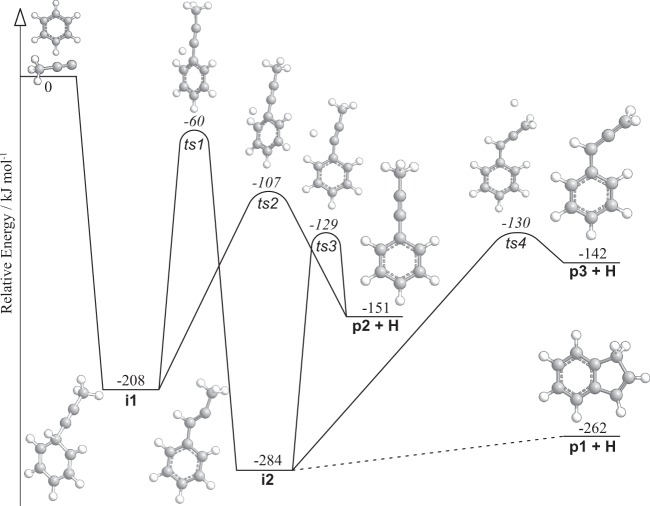
Schematic representation of the potential energy surface of the reaction of the 1-propynyl radical with benzene leading to the C_9_H_8_ isomers indene (**p1**), 1-phenyl-1-propyne (**p2**), and 1-phenyl-1,2-propadiene (**p3**). Details connecting **i2** to **p1** are found in ref.^1^.

We now combine the experimental findings with electronic structure calculations to elucidate the underlying reaction dynamics and the nature of the structural isomer(s) produced by considering three aromatic C_9_H_8_ isomers. These are indene (**p1**), 1-phenyl-1-propyne (**p2**), and 1-phenyl-1,2-propadiene (**p3**) with computed reaction energies of −262 ± 6, −151 ± 6, and −142 ± 6 kJ mol^−1^, respectively. Considering the experimentally derived reaction energy of −157 ± 27 kJ mol^−1^, the computational data support the formation of the C_9_H_8_ isomers **p2** and/or **p3** via hydrogen atom loss. Continuing on the basis of energetics, indene (**p1**) can be reasonably excluded from consideration among the detected reaction products where the formation of the indene isomer would increase the maximum translational energy beyond the error limits (±27 kJ mol^−1^) of the *P(E*_T_) to about 300 kJ mol^−1^. By examining the reaction potential energy surface (PES), we can gain deeper insight into the underlying reaction mechanism to form C_9_H_8_ isomers via the bimolecular reaction of 1-propynyl with benzene (Fig. 6; Supplemental Information).

**Figure 7:**
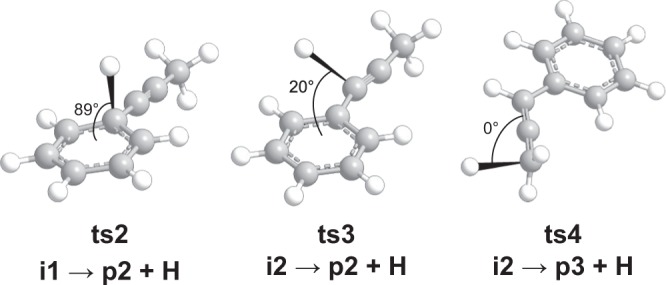
Computed geometries of the exit transition states leading to the formation of 1-phenyl-propyne (**p2**) and 1-phenyl-1,2-propadiene (**p3**). The angle for each departing hydrogen atom is given with respect to the rotational plane of the decomposing complex.

The 1-propynyl radical can add to the π-electron system of the benzene molecule without an entrance barrier (Fig. S1) forming a C_9_H_9_ intermediate **i1** featuring the propynyl substituted to the six-membered ring that lies 208 kJ mol^−1^ below the energy of the separated reactants. Intermediate **i1** can dissociate via **ts2** by eliminating the newly-formed *sp*^3^ hydrogen atom on the six-membered ring (Fig. S2) nearly perpendicular to the orbital plane of the molecule at an angle of 88.5° (Fig. 7) to form 1-phenyl-1-propyne (**p2**). The **ts2** exit transition state is characterized by the restoration of aromaticity and hence is rather tight at 44 kJ mol^−1^ above the **p2** product channel. Alternatively, the *sp*^3^ hydrogen atom on **i1** can migrate to the C1 exocyclic position over a 148 kJ mol^−1^ barrier (**ts1**) to form **i2** gaining an additional 76 kJ mol^−1^ in stability. From **i2**, each of the three C_9_H_8_ isomers **p1, p2**, and **p3** can be accessed (Fig. S2). Elimination of the exocyclic C1 or methyl hydrogen atoms results in the formation of **p2** or 1-phenyl-1,2-propadiene (**p3**) via **ts3** or **ts4**, respectively. Unlike the **i1** → **p2 + H** pathway where the hydrogen atom is ejected nearly parallel to the total angular momentum vector via **ts2**, the exit transition states **ts3** and **ts4** connecting **i2 → p2 + H** and **i2 → p3 + H** eliminate the hydrogen atom at angles of 20° and 0° with respect to the orbital plane of the molecule (Fig. 7). The minimum energy path to **p1**, not explicitly considered here since it was computed previously, involves extensive isomerization through an additional 8 intermediates and 9 transition states^1,5^.

**Figure 8:**
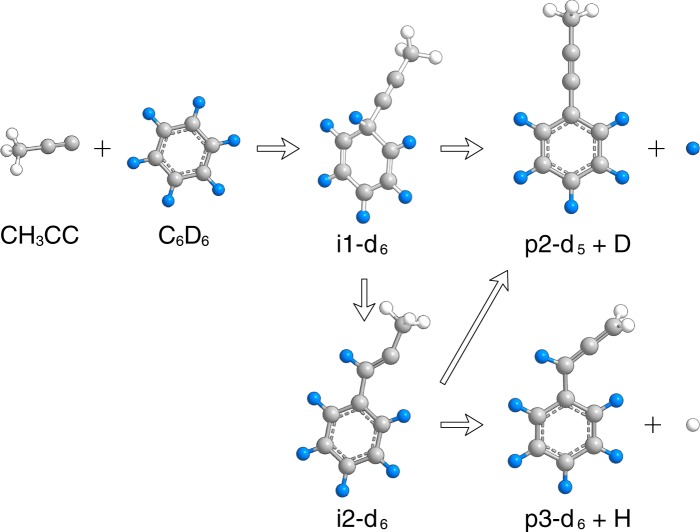
Reaction schematic for the bimolecular reaction of the 1-propynyl (CH_3_CC) radical with D6-benzene (C_6_D_6_) leading to C_9_H_3_D_5_ and C_9_H_2_D_6_ products via atomic deuterium and hydrogen loss pathways.

Although both isomers **p2** and **p3** can account for the experimental translational energy of C_9_H_8_, a close inspection of the C_9_H_9_ PES alongside our isotopically labelled study permits a clarification of the experimental reaction dynamics. The CH_3_CC – C_6_D_6_ reactive scattering experiment exposed the exclusive formation of C_9_H_3_D_5_ via elimination of atomic deuterium originating at the benzene reactant. Isotopologues of indene **p1**, if formed, would give signals for atomic hydrogen (C_9_H_2_D_6_) *and* deuterium loss (C_9_H_3_D_5_) at a ratio of about 1:1 via a decomposing bicyclic intermediate^1,5^, whereas **D6-p3** (C_6_D_5_CDCCH_2_) would form by elimination of a methyl hydrogen atom from **D6-i2**. Hence, only **D5-p2** (C_6_D_5_CCCH_3_) can account for this observation via dissociation of intermediates **D6-i1** or **D6-i2** (Fig. 8). Recalling that the best-fit *T(θ)* distribution peaked sideways at 90°, we note that of the three dissociation channels considered, only the **i1 → ts2 → p2 + **H path supports this finding where the hydrogen emitted by **ts2** occurs nearly parallel to the principal axis with the greatest moment of inertia (*I*_c_ = 1.16 × 10^−44^ kg m^2^) at an angle of 1.5°. For an asymmetric top decomposing at the top of an exit barrier, microcanonical transition state theory has shown that hydrogen atom emission from a long-lived complex along the principal axis of the greatest moment of inertia (*C*) results in sideways scattering^30,31^, as observed in this experiment (Fig. 5). The **i2 → ts3 → p2 + H** and **i2 → ts4 → p3 + H** pathways do not give rise to sideways scattering where **ts4** dissociates in the *AB* plane and **ts3** emits its hydrogen atom nearly perpendicular to its *C* axis. We also note the average *E*_T_ for C_9_H_8_ this experiment is 57 ± 8 kJ mol^−1^ and is a close match to the exit barrier of 44 ± 5 kJ mol^−1^ for the **p2** + H product channel. At the experimental collision energy of 40.8 ± 0.5 kJ mol^−1^ each of the **p1-p3** product channels are open, however, considering the barriers to isomerization on the C_9_H_9_ PES, the low energy route to 1-phenyl-1-propyne (**p2**) via intermediate **i1** is expected to dominate in a statistically-conforming molecular system, where the **i1 → i2** barrier (**ts1**) is 47 kJ mol^−1^ greater than that required for dissociation to **p2** + H via **ts2**. This is supported by our RRKM calculations which suggest that **p2** accounts for more than 99% of the total C_9_H_8_ yield. Combined, our computational analysis and experimental data indicate the formation of 1-phenyl-1-propyne (**p2**) as the predominant product formed in the bimolecular reaction of 1-propynyl with benzene via a simple addition/elimination mechanism, i.e. CH_3_CC + C_6_H_6_** → i1 → p2 + **H.

The temperature-dependent rate constant for the barrierless reaction of 1-propynyl (CH_3_CC) with benzene (C_6_H_6_) was evaluated using the long-range transition state theory, where the capture rate is assessed considering dipole-quadrupole, dipole-induced dipole and dispersion interactions between the reactants. The calculations gave the value of the rate constant increasing from 3.7 × 10^−10^ cm^3^ molecule^−1^ s^−1^ at 10 K to 5.4 × 10^−10^ and 6.5 × 10^−10^ cm^3^ molecule^−1^ s^−1^ at 100 and 300 K, respectively. These results corroborate our hypothesis that the reaction of the 1-propynyl radical with benzene should be fast even in cold molecular clouds and the rate constant can be included in astronomic kinetic models.

Lastly, we compare our results to the analogous phenyl (C_6_H_5_; X^2^A_1_) plus methylacetylene (CH_3_CCH; X^1^A_1_) reaction, where a hydrogen atom has been transferred from benzene to 1-propynyl – that, despite its obvious similarity with the title reaction, follows completely distinct reaction dynamics. In a low-pressure (300 Torr) high temperature (1200–1500 K) chemical reactor the phenyl reacts with methylacetylene to form the C_9_H_8_ isomers indene (**p1**), 1-phenyl-1-propyne (**p2**), 1-phenyl-1,2-propadiene (**p3**) at fractions of 10, 82, and 8% respectively^1^; in a high-energy (*E*_C_ = 140 kJ mol^−1^) crossed molecular beams experiment, a similar outcome was observed with the formation of 1-phenyl-1-propyne (**p2**) via an addition/elimination reaction mechanism via a short-lived reaction adduct^8^. In the low-energy (*E*_C_ = 45 kJ mol^−1^) case, however, the C_6_H_5_ plus CH_3_CCH reaction results in the formation of indene (**p1**) as confirmed through a series of isotopic experiments and supported by RRKM calculations in the zero-pressure limit suggesting the indene isomer accounted for 81–91% of all hydrogen-loss products formed^5^. Although the 1-phenyl-1-propyne (**p2**) isomer was predicted to occur at a level of 7–10%, it could not be explicitly traced in the crossed molecular beam experiments. The most recent theory describing the C_6_H_5_ plus CH_3_CCH reaction suggests the phenyl addition to methylacetylene is inhibited by at least a 6 kJ mol^−1^ entrance barrier^1^, and results in the formation of the geometric isomer of **i2** with the methyl-group set *trans* to the phenyl ring that undergoes *cis-trans* isomerization to **i2** via a barrier of only 20 kJ mol^−1^; all remaining transition states are energetically lower than the separated reactants and the system proceeds spontaneously to indene (**p1**) via annulation at the phenyl moiety. Isomerization to **i1**, the initial intermediate formed in the title reaction, is inhibited by a rather large barrier of 221 kJ mol^−1^ and thus is not competitive with the low-energy path to indene formation. Hence the nature of the entrance channel (barrier vs barrierless), initial intermediate formed, and the high-energy **i1–i2** transition state (**ts1**) underlie the distinct reaction dynamics followed in the analogous CH_3_CC + C_6_H_6_ and CH_3_CCH + C_6_H_5_ reactions under similar experimental conditions.

## Conclusion

Crossed molecular beams (CMB) experiments, combined with electronic structure and statistical calculations, were exploited to investigate the reaction dynamics of the of 1-propynyl (CH_3_CC; X^2^A_1_) reaction with benzene (C_6_H_6_; X^1^A_1g_) under single-collision conditions. The reaction was found to proceed indirectly and is initiated by the *barrierless* addition of 1-propynyl to a carbon atom on the benzene ring forming a low-lying intermediate **i1** that eliminates the hydrogen atom to form 1-phenyl-1-propyne (C_6_H_5_CCCH_3_; **p2**) in an overall exoergic reaction (experimental, −157 ± 27 kJ mol^−1^; computational, −151 ± 6 kJ mol^−1^). Our RRKM analysis suggests that this pathway should account for more than 99% of C_9_H_8_ products. Interestingly, differences in the entrance channel for the title reaction as compared to phenyl (C_6_H_5_) addition to methylacetylene (CH_3_CCH) give rise to distinct reaction products under similar experimental conditions, with the latter forming the PAH indene (C_9_H_8_; **p1**) as the major hydrogen-loss product. Compared to prior experiments probing access to the C_9_H_9_ potential energy surface, such as with phenyl addition to methylacetylene and benzyl (C_6_H_5_CH_2_) addition to acetylene (C_2_H_2_), the reaction mechanism uncovered here is distinguished by being the first barrierless path to C_9_H_8_ where the 1-propynyl radical can disrupt aromaticity at benzene without a penalty. Despite the advantage, this pathway is not likely to compete with PAH producing reaction schemes in high temperature combustion environments where the availability of 1-propynyl radicals are likely linked to the decomposition of and/or hydrogen abstraction from the hydrocarbon methylacetylene. Under these conditions, methylacetylene is more likely to – in the case of H atom reactions – form allene via hydrogen-assisted isomerization, or react producing acetylene by methyl displacement or the propargyl radical (CH_2_CCH) by hydrogen abstraction, rather than form 1-propynyl by elimination or abstraction of its acetylenic hydrogen atom^32,33^.

The isolobal ethynyl (C_2_H; X^2^Σ^+^) radical, where the methyl group has been replaced by a hydrogen atom, presents an analogous chemistry to that uncovered for the 1-propynyl radical. Using the D1-ethynyl (C_2_D) and D6-benzene isotopologues, molecular beams experiments demonstrated the formation of D6-phenylacetylene (C_6_D_5_CCD) in a mechanism similar to the title reaction where dissociation immediately follows the barrierless formation of a low-lying C_8_D_7_ intermediate^34,35^. In each case, the reactivity is localized to the radical end of the alkyne, rendering the D/CH_3_ substituents mere spectators in the reaction coordinate. The addition/elimination reaction mechanism via a (pseudo)barrierless entrance channel is also observed in the isoelectronic boronyl (BO; X^2^Σ^+^)-benzene and cyano (CN; X^2^Σ^+^)-benzene reaction systems. Addition of the boronyl radical followed by hydrogen atom loss results in the overall exoergic formation of phenyl oxoborane (C_6_H_5_BO)^36,37^; also nearly 20 years before its detection in the interstellar medium^38^, molecular beams experiments demonstrated that cyanobenzene (C_6_H_5_CN) is formed via a low-energy, barrier-less pathway involving two neutral reactants that, in theory, proceeds spontaneously at 0 K and proposed the aromatic molecule to be present in cold molecular clouds^39,40^.

Although the 1-propynyl radical has not yet been detected in astrophysical sources, methylacetylene – a potential precursor – is both abundant and prolific in different regions of space^41,42^, and could provide a source of 1-propynyl radicals in the photodissociation region of molecular clouds or near carbon-rich AGB stars via photolytic cleavage of the acetylenic C–H bond (CH_3_CCH + hν → CH_3_CC + H)^24–26^. To establish the activity of the 1-propynyl radical in astrochemistry, laboratory characterizations of its structure are needed to aid observation efforts toward its detection and ultimately its inclusion in astrochemical modelling networks alongside its low-energy isomer, propargyl (CH_2_CCH). Considering that all barriers are below the energy of the separated reactants, 1-propynyl addition to benzene is a plausible reaction scheme for the low temperature extremes that persist in cold molecular clouds and strongly suggests the existence of 1-phenyl-1-propyne (C_6_H_5_CCCH_3_) in molecular clouds, though additional spectroscopic data are required to support its assignment among existing astronomical data^43^.

## Methods

### Experimental

The reactions of 1-propynyl (CH_3_CC; X^2^A_1_) with benzene (C_6_H_6_; X^1^A_1g_) and D6-benzene (C_6_D_6_; X^1^A_1g_) were carried out under single collision conditions exploiting a universal crossed molecular beams machine at the University of Hawaii^44–49^. The pulsed 1-propynyl radical beam was produced by photodissociation of 1-iodopropyne (CH_3_CCI; TCI, 99%+) seeded at a level of 0.5% in helium (99.9999%; AirGas) at 193 nm (Complex 110, Coherent, Inc.) at 30 Hz and 20 mJ per pulse in the primary source chamber^50–53^. This gas mixture was stored in a Teflon-coated sample cylinder and was introduced into a piezoelectric pulsed valve operating at 60 Hz, pulse widths of 80 μs, peak voltages of −400 V, and at 760 Torr backing pressure. The pulsed 1-propynyl beam passes through a skimmer and is then velocity selected by a four-slot chopper wheel rotating at 120 Hz. On-axis (Θ = 0°) characterization of the primary beam determines a peak velocity *v*_p_ of 1658 ± 12 m s^−1^ and speed ratio *S* of 7.1 ± 0.3. This section of the primary beam collides perpendicularly with a supersonic beam of (D6)-benzene prepared in the secondary source chamber. A pulsed valve in the secondary source operated at a repetition rate of 60 Hz, a pulse width of 80 μs, and a peak voltage of −400 V, generated a pulsed molecular beam of (D6)-benzene [Aldrich Chemistry; ≥99.9% (>99.9% atom D)] seeded in argon (99.9999%, AirGas) at fractions of 10% at 550 Torr. The (D6)-benzene peak velocities were determined to be *v*_p_ = 622 ± 10 ms^−1^ with *S* = 19.3 ± 0.6 (*v*_p_ = 623 ± 9 ms^−1^, *S* = 19.3 ± 0.4) resulting in a nominal collision energy *E*_C_ of 40.8 ± 0.5 kJ mol^−1^ (41.8 ± 0.6 kJ mol^−1^) and centre-of-mass angle Θ_CM_ of 36.9 ± 0.5° (39.0 ± 0.5°) (Table 1). We note that any propargyl (CH_2_CCH; X^2^B_1_) radicals formed in the photodissociation of 1-iodopropyne as the result of isomerization from 1-propynyl do not react under our experimental conditions with benzene, where addition to benzene is inhibited by a barrier of 65–75 kJ mol^−1^ ^54^; this barrier cannot be overcome at collision energies of 40.8 ± 0.5 kJ mol^−1^ in our present experiments.

**Table 1:** Peak velocities (*v*_p_) and speed ratios (*S*) of the 1-propynyl (CH_3_CC), benzene (C_6_H_6_), and D6-benzene (C_6_D_6_) beams along with the corresponding collision energies (*E*_C_) and center-of-mass angles (Θ_CM_).

Beam	*v*_p_ (m s^−1^)	*S*	*E*_C_ (kJ mol^−1^)	Θ_CM_ (degree)
CH_3_CC (X^2^A_1_)	1658 ± 12	7.1 ± 0.3		
C_6_H_6_ (X^1^A_1g_)	622 ± 10	19.3 ± 0.6	40.8 ± 0.5	36.9 ± 0.5
C_6_D_6_ (X^1^A_1g_)	623 ± 9	19.3 ± 0.4	41.8 ± 0.6	39.0 ± 0.5

The crossed molecular beams machine employs two beam sources affixed at 90 degrees and a triply differentially pumped universal detector that is rotatable in the plane defined by the reactant beams^55^. The detector houses an electron impact ionizer coupled to a quadrupole mass spectrometer that permits filtering of ionized (80 eV) products according to mass-to-charge ratio. Ions are ultimately registered by a photomultiplier tube and filed into bins by multichannel scaling according to time of arrival to produce a time-of-flight (TOF) spectrum. The laboratory data are forward-convoluted to the centre-of-mass (CM) frame and the resulting CM translational energy *P(E*_T_*)* and angular *T(θ)* flux distributions are analysed to inform the reaction dynamics^56,57^. Errors of the *P(E*_T_*)* and *T(θ)* functions are determined within the 1σ error limits of the accompanying LAB angular distribution while maintaining a good fit of the laboratory TOF spectra.

### Theoretical

Geometries of the reactants, intermediates, transition states, and products of the CH_3_CC - C_6_H_6_ system were optimized at the density functional B3LYP/6-311 G(d,p) level of theory^58,59^. Vibrational frequencies were computed at the same theoretical level and were used for the evaluation of zero-point vibrational energy corrections (ZPE) and in calculations of rate constants. Energies were refined by single-point calculations using the model chemistry G3(MP2,CC)//B3LYP/6-311 G(d,p) level of theory^60–62^. This composite approach normally provides a chemical accuracy of 3–6 kJ mol^–1^ for the relative energies and 0.01–0.02 Å for bond lengths as well as 1–2° for bond angles^62^. The ab initio calculations were performed using the GAUSSIAN 09^63^ and MOLPRO 2010^64^ program packages. Rate constants of all pertinent unimolecular reaction steps on the C_9_H_9_ PES following initial association of 1-propynyl with benzene were computed using Rice-Ramsperger-Kassel-Marcus (RRKM) theory^65–67^, as functions of available internal energy of each intermediate or transition state, where numbers and densities of states were obtained within the harmonic approximation using B3LYP/6–311 G(d,p) computed frequencies. The internal energy was taken as a sum of the collision energy and a negative of the relative energy of a species with respect to the reactants (the chemical activation energy). One energy level was considered throughout as at a zero-pressure limit. Then, RRKM rate constants were utilized to compute product branching ratios by solving first-order kinetic equations within steady-state approximation^68^ using a newly developed computer code^52^. Dipole and quadrupole moments and isotropic polarizabilities of CH_3_CC and C_6_H_6_ required for the long-range transition state theory^69^ calculations of the entrance channel rate constant were computed at the density functional ωB97XD level of theory^70^ with Dunning’s correlation-consistent cc-pVTZ basis set^71^.

## Supplementary information

Supplemental Information

## Data Availability

The datasets generated during and/or analysed during the current study are available from the corresponding authors on reasonable request.

## References

[1] Zhang F (2011). VUV photoionization study of the formation of the indene molecule and its isomers. J. Phys. Chem. Lett..

[2] Kaiser RI, Asvany O, Lee YT (2000). Crossed beam investigation of elementary reactions relevant to the formation of polycyclic aromatic hydrocarbon (PAH)-like molecules in extraterrestrial environments. Planet. Space Sci..

[3] Kaiser RI, Yamada M, Osamura Y (2002). A crossed beam and ab initio investigation of the reaction of hydrogen sulfide, H_2_S(XA_1_), with dicarbon molecules, C_2_(X^1^Σ_g_^+^). J. Phys. Chem. A.

[4] Kaiser RI (2003). Elementary reactions of the phenyl radical, C_5_H_5_, with C_3_H_4_ isomers, and of benzene, C_6_H_6_, with atomic carbon in extraterrestrial environments. Astron. Astrophys..

[5] Parker DDSN, Zhang DF, Kaiser DRI, Kislov DVV, Mebel DAM (2011). Indene formation under single-collision conditions from the reaction of phenyl radicals with allene and methylacetylene—a crossed molecular beam and ab initio study. Chem. - Asian J..

[6] Parker DSN, Kaiser RI, Kostko O, Ahmed M (2015). Selective formation of indene through the reaction of benzyl radicals with acetylene. ChemPhysChem.

[7] Hansen N, Schenk M, Moshammer K, Kohse-Höinghaus K (2017). Investigating repetitive reaction pathways for the formation of polycyclic aromatic hydrocarbons in combustion processes. Combust. Flame.

[8] Kaiser RI (2000). Crossed beam reaction of phenyl radicals with unsaturated hydrocarbon molecules. I. Chemical dynamics of phenylmethylacetylene (C_6_H_5_CCCH_3_;X^1^A′) formation from reaction of C_6_H_5_(X ^2^A_1_) with methylacetylene, CH_3_CCH(X^1^A_1_). J. Chem. Phys..

[9] Vereecken L (2002). Reaction of phenyl radicals with propyne. J. Am. Chem. Soc..

[10] Gu X, Zhang F, Guo Y, Kaiser RI (2007). Reaction dynamics of phenyl radicals (C_6_H_5_, X^2^A′) with methylacetylene (CH_3_CCH(XA_1_)), allene (H_2_CCCH_2_(X^1^A_1_)), and their D4-isotopomers. J. Phys. Chem. A.

[11] Gu X, Kaiser RI (2008). Reaction dynamics of phenyl radicals in extreme environments: a crossed molecular beam study. Acc. Chem. Res..

[12] Yuan W (2017). Experimental and kinetic modeling study of premixed n-butylbenzene flames. Proc. Combust. Inst..

[13] Moshammer K (2017). Aromatic ring formation in opposed-flow diffusive 1,3-butadiene flames. Proc. Combust. Inst..

[14] Ruwe L, Moshammer K, Hansen N, Kohse-Höinghaus K (2018). Influences of the molecular fuel structure on combustion reactions towards soot precursors in selected alkane and alkene flames. Phys. Chem. Chem. Phys..

[15] Martin, J. W. *et al*. Polar curved polycyclic aromatic hydrocarbons in soot formation. *Proc. Combust. Inst*. (2018).

[16] Wu X-Z (2016). Formation of curvature subunit of carbon in combustion. J. Am. Chem. Soc..

[17] Lovas FJ (2005). Interstellar Chemistry:  A strategy for detecting polycyclic aromatic hydrocarbons in space. J. Am. Chem. Soc..

[18] Parker DSN, Kaiser RI, Troy TP, Ahmed M (2014). Hydrogen abstraction/acetylene addition revealed. Angew. Chem., Int. Ed..

[19] Kaiser RI (2002). Experimental investigation on the formation of carbon-bearing molecules in the interstellar medium via neutral–neutral reactions. Chem. Rev..

[20] Stahl F (2001). Reaction of the ethynyl radical, C_2_H, with methylacetylene, CH_3_CCH, under single collision conditions: implications for astrochemistry. J. Chem. Phys..

[21] Kaiser RI (2001). Chemical dynamics of D1-methyldiacetylene (CH_3_CCCCD; X^1^A_1_) and D1-ethynylallene (H_2_CCCH(C_2_D); X^1^A′) formation from reaction of C_2_D(X^2^Σ^+^) with methylacetylene, CH_3_CCH(X^1^A_1_). J. Chem. Phys..

[22] Kaiser RI, Stranges D, Lee YT, Suits AG (1997). Neutral-neutral reactions in the interstellar medium. I. Formation of carbon hydride radicals via reaction of carbon atoms with unsaturated hydrocarbons. Astrophys. J..

[23] Irvine WM, Hoglund B, Friberg P, Askne J, Ellder J (1981). The increasing chemical complexity of the Taurus dark clouds: detection of CH_3_CCH and C_4_H. Astrophys. J..

[24] Harich S, Lin JJ, Lee YT, Yang X (2000). Photodissociation dynamics of propyne at 157 nm. J. Chem. Phys..

[25] Sun W, Yokoyama K, Robinson JC, Suits AG, Neumark DM (1999). Discrimination of product isomers in the photodissociation of propyne and allene at 193 nm. J. Chem. Phys..

[26] Ganot Y, Rosenwaks S, Bar I (2004). H and D release in ~243.1 nm photolysis of vibrationally excited 3ν_1_, 4ν_1_, and 4ν_CD_ overtones of propyne-d_3_. J. Chem. Phys..

[27] Jones BM (2011). Formation of benzene in the Interstellar Medium. Proc. Natl. Acad. Sci. USA.

[28] Levine, R. D. *Molecular Reaction Dynamics*. (Cambridge University Press: Cambridge, U.K., 2005).

[29] Miller WB, Safron SA, Herschbach DR (1967). Exchange reactions of alkali atoms with alkali halides: A collision complex mechanism. Discuss. Faraday Soc..

[30] Smith DJ, Grice R (1991). Angular distributions of reactive scattering arising from persistent complexes with asymmetric top transition states. Mol. Phys..

[31] Grice R (1995). Dynamics of persistent collision complexes in molecular beam reactive scattering. Int. Rev. Phys. Chem..

[32] Ryazantsev MN, Jamal A, Maeda S, Morokuma K (2015). Global investigation of potential energy surfaces for the pyrolysis of C1-C3 hydrocarbons: toward the development of detailed kinetic models from first principles. Phys. Chem. Chem. Phys..

[33] Rosado-Reyes CM, Manion JA, Tsang W (2010). Kinetics of the thermal reaction of H atoms with propyne. J. Phys. Chem. A.

[34] Jones B, Zhang F, Maksyutenko P, Mebel AM, Kaiser RI (2010). Crossed molecular beam study on the formation of phenylacetylene and its relevance to Titan’s atmosphere. J. Phys. Chem. A.

[35] Gu X, Zhang F, Guo Y, Kaiser RI (2007). Crossed-molecular-beam study on the formation of phenylacetylene from phenyl radicals and acetylene. Angew. Chem., Int. Ed..

[36] Kaiser RI, Balucani N (2017). Exploring the gas phase synthesis of the elusive class of boronyls and the mechanism of boronyl radical reactions under single collision conditions. Acc. Chem. Res..

[37] Parker DSN (2013). Gas-phase synthesis of phenyl oxoborane (C_6_H_5_BO) via the reaction of boron monoxide with benzene. J. Org. Chem..

[38] McGuire BA (2018). Detection of the aromatic molecule benzonitrile (C_6_H_5_CN) in the interstellar medium. Science.

[39] Balucani N (1999). Crossed beam reaction of cyano radicals with hydrocarbon molecules. I. Chemical dynamics of cyanobenzene (C_6_H_5_CN; X^1^A_1_) and perdeutero cyanobenzene (C_6_D_5_CN; X^1^A_1_) formation from reaction of CN(X^2^Σ^+^) with benzene C_6_H_6_(X^1^A_1g_), and D6-benzene C_6_D_6_(X^1^A_1g_). J. Chem. Phys..

[40] Kaiser RI, Balucani N (2001). The formation of nitriles in hydrocarbon-rich atmospheres of planets and their satellites:  laboratory investigations by the crossed molecular beam technique. Acc. Chem. Res..

[41] Guzmán VV (2014). Chemical complexity in the horsehead photodissociation region. Faraday Discuss..

[42] Gratier P (2016). A new reference chemical composition for TMC-1. Astrophys. J., Suppl. Ser..

[43] Powers DE, Hopkins JB, Smalley RE (1981). Vibrational relaxation in jet-cooled phenylalkynes. J. Chem. Phys..

[44] Kaiser RI, Balucani N, Charkin DO, Mebel AM (2003). A crossed beam and ab initio study of the C_2_(X^1^Σ_g_^+^/a^3^Π_u_) + C_2_H_2_(X^1^Σ_g_^+^) reactions. Chem. Phys. Lett..

[45] Balucani N, Asvany O, Kaiser RI, Osamura Y (2002). Formation of three C_4_H_3_N isomers from the reaction of CN (X^2^Σ^+^) with allene, H_2_CCCH_2_ (XA_1_), and methylacetylene, CH_3_CCH (X^1^A_1_):  a combined crossed beam and ab initio study. J. Phys. Chem. A.

[46] Kaiser RI, Mebel AM, Chang AHH, Lin SH, Lee YT (1999). Crossed-beam reaction of carbon atoms with hydrocarbon molecules. V. Chemical dynamics of n-C_4_H_3_ formation from reaction of C(^3^P_j_) with allene, H_2_CCCH_2_(X ^1^A_1_). J. Chem. Phys..

[47] Kaiser RI (1999). Crossed beams reaction of atomic carbon, C(^3^P_j_), with D6-benzene, C_6_D_6_(X ^1^A_1g_): observation of the per-deutero-1,2-didehydro-cycloheptatrienyl radical, C_7_D_5_(X^2^B_2_). J. Chem. Phys..

[48] Balucani N, Mebel AM, Lee YT, Kaiser RI (2001). A combined crossed molecular beam and ab initio study of the reactions C_2_(X^1^Σ_g_^+^, a^3^Π_u_) + C_2_H_4_ → *n*-C_4_H_3_(X^2^Aʹ) + H(^2^S_1/2_). J. Phys. Chem. A.

[49] Kaiser RI (2010). Untangling the chemical evolution of Titan’s atmosphere and surface - from homogeneous to heterogeneous chemistry. Faraday Discuss..

[50] Thomas AM, Zhao L, He C, Mebel AM, Kaiser RI (2018). A combined experimental and computational study on the reaction dynamics of the 1-propynyl (CH_3_CC)–acetylene (HCCH) system and the formation of methyldiacetylene (CH_3_CCCCH). J. Phys. Chem. A.

[51] Thomas AM (2019). Combined experimental and computational study on the reaction dynamics of the 1-propynyl (CH_3_CC)–1,3-butadiene (CH_2_CHCHCH_2_) system and the formation of toluene under single collision conditions. J. Phys. Chem. A.

[52] He C (2019). Elucidating the chemical dynamics of the elementary reactions of the 1-propynyl radical (CH_3_CC; X^2^A_1_) with methylacetylene (H_3_CCCH; X^1^A_1_) and allene (H_2_CCCH_2_; X^1^A_1_). J. Phys. Chem. A.

[53] He C, Thomas AM, Galimova GR, Mebel A, Kaiser RI (2019). Gas phase formation of methyltriacetylene (CH_3_(C≡C)_3_H) - an interstellar molecule. ChemPhysChem.

[54] Kislov VV, Mebel AM (2007). Ab initio G3-type/statistical theory study of the formation of indene in combustion flames. I. Pathways involving benzene and phenyl radical. J. Phys. Chem. A.

[55] Kaiser RI, Mebel AM, Lee YT (2001). Chemical dynamics of cyclopropynylidyne (c-C_3_H; X^2^B_2_) formation from the reaction of C(^1^D) with acetylene, C_2_H_2_(X^1^Σ_g_^+^). J. Chem. Phys..

[56] Gu X, Guo Y, Zhang F, Mebel AM, Kaiser RI (2006). Reaction dynamics of carbon-bearing radicals in circumstellar envelopes of carbon atars. Faraday Discuss..

[57] Huang LCL, Balucani N, Lee YT, Kaiser RI, Osamura Y (1999). Crossed beam reaction of the cyano radical, CN(X ^2^Σ^+^), with methylacetylene, CH_3_CCH (X ^1^A_1_): observation of cyanopropyne, CH_3_CCCN (X ^1^A_1_), and cyanoallene, H_2_CCCHCN (X ^1^A′). J. Chem. Phys..

[58] Becke AD (1993). Density-functional thermochemistry. III. The role of exact exchange. J. Chem. Phys..

[59] Lee C, Yang W, Parr RG (1988). Development of the Colle-Salvetti correlation-energy formula into a functional of the electron density. Phys. Rev. B.

[60] Adler TB, Knizia G, Werner H-J (2007). A simple and efficient CCSD(T)-F12 approximation. J. Chem. Phys..

[61] Knizia G, Adler TB, Werner H-J (2009). Simplified CCSD(T)-F12 methods: theory and benchmarks. J. Chem. Phys..

[62] Curtiss LA, Raghavachari K, Redfern PC, Baboul AG, Pople JA (1999). Gaussian-3 theory using coupled cluster energies. Chem. Phys. Lett..

[63] Frisch, M. *et al*. *GAUSSIAN 09, revision A.1* (Gaussian Inc.: Wallingford, CT, 2009).

[64] Werner, H.-J. *et al*. *MOLPRO, Version 2010.1, A package of ab initio programs*, University of Cardiff: Cardiff, UK, see http://www.molpro.net (2010).

[65] Robinson, P. J. & Holbrook, K. A. *Unimolecular Reactions*. (John Wiley & Sons, Ltd.: New York, NY, 1972).

[66] Eyring, H., Lin, S. H. & Lin, S. M. *Basic Chemical Kinetics*. (John Wiley and Sons, Inc.: New York, NY, 1980).

[67] Steinfield, J., Francisco, J. & Hase, W. *Chemical Kinetics and Dynamics*. (Prentice Hall: Englewood Cliffs, NJ, 1982).

[68] Kislov VV, Nguyen TL, Mebel AM, Lin SH, Smith SC (2004). Photodissociation of benzene under collision-free conditions: an ab initio/Rice-Ramsperger-Kassel-Marcus study. J. Chem. Phys..

[69] Georgievskii Y, Klippenstein SJ (2005). Long-range transition state theory. J. Chem. Phys..

[70] Chai J-D, Head-Gordon M (2008). Long-range corrected hybrid density functionals with damped atom-atom dispersion corrections. Phys. Chem. Chem. Phys..

[71] Dunning TH (1989). Gaussian basis sets for use in correlated molecular calculations. I. The atoms boron through neon and hydrogen. J. Chem. Phys..

