# Supplementary material for: A Barrierless Pathway Accessing the C9H9 and C9H8 Potential Energy Surfaces via the Elementary Reaction of Benzene with 1-Propynyl
Source: Sci Rep. 2019 Nov 26;9:17595. doi: 10.1038/s41598-019-53987-5 (PMC6879741; doi:10.1038/s41598-019-53987-5)
Supplement: Supplementary file 1 — Supplemental Information [file 41598_2019_53987_MOESM1_ESM.pdf]

SUPPLEMENTAL INFORMATION

A Barrierless Pathway Accessing the C_9H_9 and C_9H_8 Potential Energy Surfaces via the Elementary Reaction of Benzene with 1-Propynyl

Aaron M. Thomas, Srinivas Doddipatla, Ralf I. Kaiser*

Department of Chemistry, University of Hawai'i at Manoa, Honolulu, Hawaii 96822, United States

Galiya R. Galimova,[1,2] Alexander M. Mebel[1,2]*

[1] Samara National Research University, Samara 443086, Russia and

[2] Department of Chemistry and Biochemistry, Florida International University, Miami, Florida 33199, United States

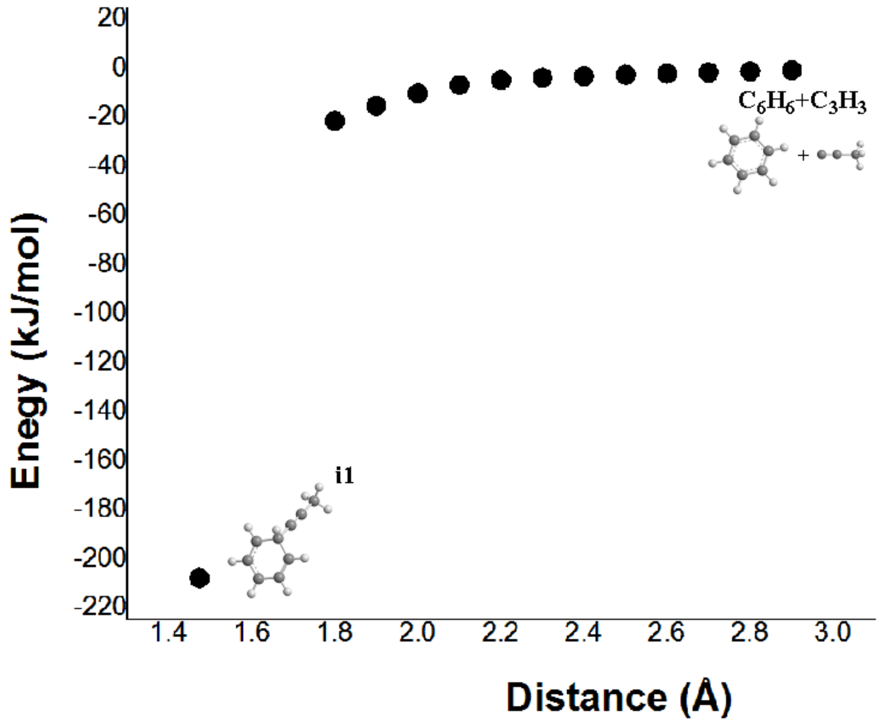

Figure S1. Minimal energy reaction path from the 1-propynyl + benzene reactants to intermediate **i1** along the R(C-C) distance for the forming C-C bond calculated at the B3LYP/6-311G(d,p) level of theory.

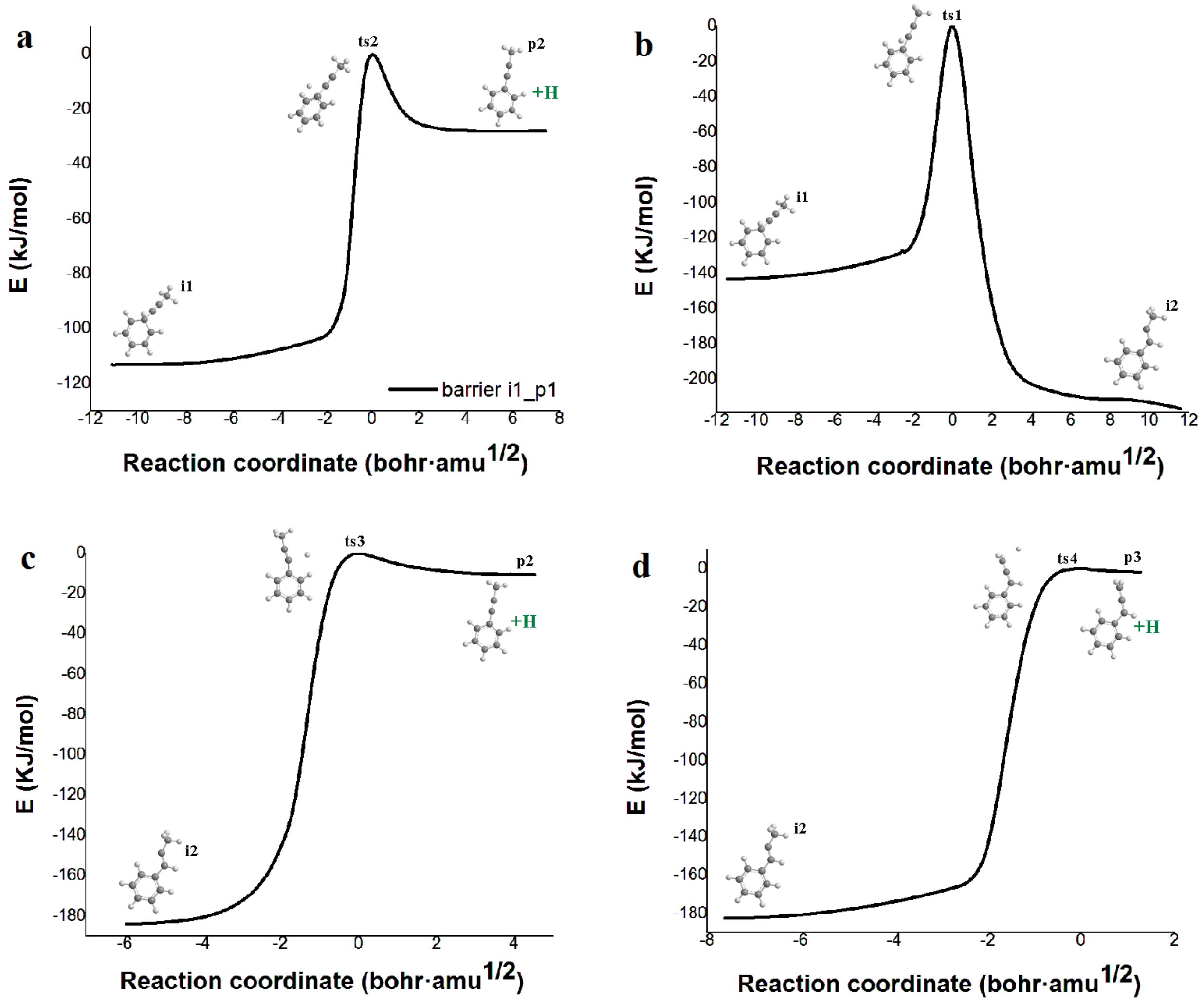

Figure S2. Results of IRC calculations at the B3LYP/6-311G(d,p) level of theory for transition states involved in the 1-propynyl + benzene reaction. For each transition state relative energies are shown with respect to this particular transition state.

Table S1. Optimized Cartesian coordinates and vibrational frequencies of various species involved in the 1-propynyl + benzene reaction.

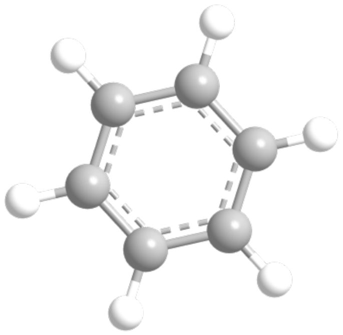

C_6H_6, D_{6h}, $^1A_1$

Cartesian Coordinates

C	0.416863	1.330014	0.000000
C	1.360530	0.304248	0.000000
C	0.943545	-1.026058	0.000000
C	-0.416717	-1.330074	0.000000
C	-1.360541	-0.304183	0.000000
C	-0.943613	1.026027	0.000000
H	0.740511	2.365049	0.000000
H	2.418883	0.540426	0.000000
H	1.677832	-1.824055	0.000000
H	-0.741305	-2.364808	0.000000
H	-2.418808	-0.540822	0.000000
H	-1.677523	1.824364	0.000000

Vibrational Frequencies

412.9072	413.7916	623.2856
623.4868	689.0140	723.3250
862.5482	862.8397	981.3337
982.0883	1012.9151	1016.8790
1023.3623	1060.0209	1060.5558
1174.5777	1197.1803	1197.7243
1334.5913	1381.6540	1512.6212
1513.1645	1636.9732	1637.2256
3155.2009	3164.5501	3165.1141
3180.3081	3180.9177	3191.2119

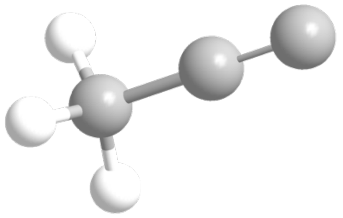

CH_3CC, C_{3v}, $^2A_1$

Cartesian Coordinates

H	0.000000	1.021621	-1.512622
C	0.000000	0.000000	-1.133496
C	0.000000	0.000000	0.336404
H	0.884750	-0.510811	-1.512622
H	-0.884750	-0.510811	-1.512622
C	0.000000	0.000000	1.553404

Vibrational Frequencies

125.	125.	934.
1009.	1009.	1410.
1479.	1479.	2190.
3054.	3133.	3133.

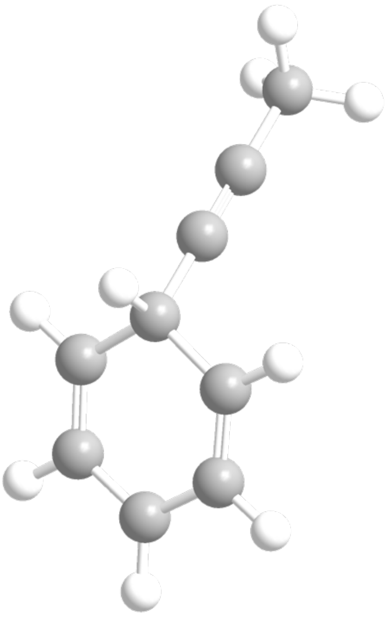

i1, C_s, $^2A'$

Cartesian Coordinates

C	-0.790737	-1.259583	0.370452
C	-0.062465	-0.000044	0.792398
C	-0.790675	1.259552	0.370525
C	-1.994496	1.224242	-0.263937
C	-2.630063	0.000054	-0.588194
C	-1.994568	-1.224179	-0.263991
H	-0.306794	-2.201808	0.599615
H	-0.012457	-0.000078	1.899184
H	-0.306698	2.201750	0.599732
H	-2.476867	2.157551	-0.536580
H	-3.586539	0.000094	-1.095543
H	-2.476997	-2.157451	-0.536659
C	1.335807	-0.000074	0.325032
C	2.478447	-0.000116	-0.052971
C	3.859879	0.000021	-0.520215
H	4.055244	-0.857750	-1.170513
H	4.081786	0.906706	-1.090766
H	4.562557	-0.048268	0.316934

Vibrational Frequencies

12.6309	56.1326	91.7663
173.2970	280.7581	309.3727
386.5748	416.1430	507.3568
525.1549	590.3887	610.5900
646.2964	738.7899	768.3601
833.8136	878.9039	966.9152
972.7417	983.9553	990.3358
1018.9507	1054.2840	1055.9433
1114.0271	1162.7890	1170.7659
1197.7592	1269.1637	1276.2378
1343.1405	1393.1601	1417.0596
1447.8102	1480.5127	1481.4814
1539.8354	1601.1642	2352.6324
2869.4790	3021.4059	3077.6049
3078.4514	3154.7063	3157.3374
3182.0244	3182.4917	3196.2112

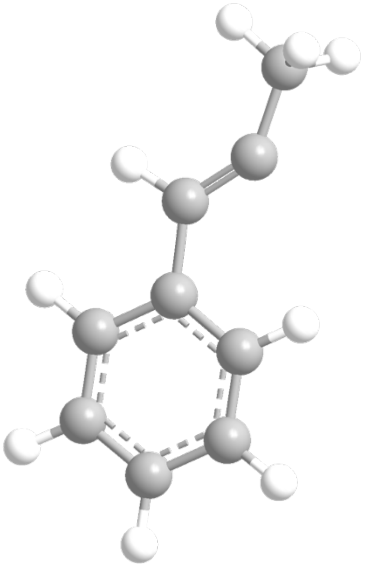

i2, C_1, ^2A

Cartesian Coordinates

C	-0.267776	-0.242398	-0.000119
C	1.163517	-0.590133	-0.000168
C	2.188761	0.235690	0.000850
C	3.654646	0.209129	0.000032
C	-0.710998	1.090235	-0.000127
C	-2.067205	1.387261	0.000012
C	-3.015313	0.362145	0.000214
C	-1.230054	-1.261456	0.000003
C	-2.590328	-0.963444	0.000223
H	1.380242	-1.666647	-0.000586
H	4.028031	-0.826949	0.002402
H	4.064736	0.709109	-0.883835
H	4.066050	0.713585	0.880711
H	0.022426	1.889004	-0.000315
H	-2.390204	2.422680	0.000049
H	-4.073590	0.597438	0.000260
H	-0.905522	-2.297208	0.000087
H	-3.317426	-1.768041	0.000306

Vibrational Frequencies

36.1243	98.9347	144.8289
197.2379	271.3194	376.6139
416.0018	448.6868	497.6363
628.3745	636.7143	709.7803
742.9035	797.0675	830.5471
852.5702	927.0649	929.8777
976.2154	998.5649	1015.2735
1020.5060	1034.9807	1050.5717
1104.4123	1181.5913	1201.6797
1214.4838	1277.8890	1342.6090
1360.4677	1394.2245	1456.8825
1470.9990	1480.3645	1523.6077
1616.8740	1639.1411	1736.8256
2972.6728	3038.7679	3056.8852
3073.4863	3156.5483	3163.5235
3173.6059	3185.7514	3194.2567

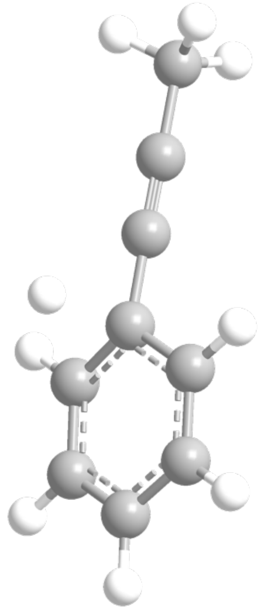

ts2, C_s, $^2A'$

Cartesian Coordinates

C	0.035600	-0.000025	0.200080
C	-1.398275	-0.000043	0.089354
C	-2.597037	0.000004	-0.030221
C	-4.047697	0.000031	-0.163753
C	0.757363	-1.220614	0.039281
C	2.133950	-1.209295	-0.092162
C	2.834283	0.000035	-0.139656
C	0.757309	1.220597	0.039290
C	2.133898	1.209333	-0.092156
H	-0.009576	-0.000072	1.958959
H	-4.480875	-0.909648	0.262169
H	-4.489608	0.855554	0.355353
H	-4.349250	0.053950	-1.214253
H	0.209376	-2.154179	0.064549
H	2.670672	-2.147881	-0.174311
H	3.913033	0.000058	-0.243119
H	0.209272	2.154131	0.064605
H	2.670584	2.147941	-0.174288

Vibrational Frequencies

1025.6876*i*	8.3139	91.8813
93.8603	228.3820	296.7694
358.8895	394.8927	415.5431
476.7098	517.8976	537.4942
561.5368	629.9958	680.9646
715.0448	781.7555	835.3001
917.6228	975.8445	976.6525
994.6642	1009.7265	1040.3740
1052.1650	1053.9404	1100.0689
1178.2441	1197.2852	1280.4501
1288.5194	1347.6528	1416.6387
1465.0404	1478.3355	1480.0804
1502.5245	1576.7182	1621.6219
2347.7038	3021.4602	3076.8384
3081.0973	3164.8839	3172.1937
3187.0736	3195.3908	3198.6448

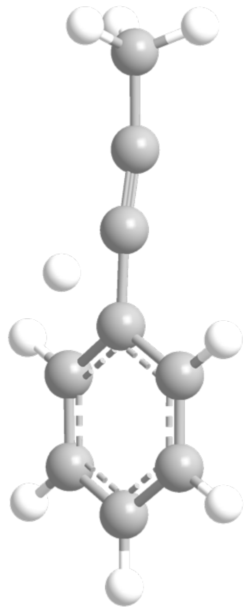

ts1, C$_s$, ^{2}A'

Cartesian Coordinates

C	-0.047328	-0.000016	0.094693
C	1.397766	-0.000027	0.066986
C	2.606399	-0.000012	-0.119731
C	4.061234	0.000010	-0.066357
C	-0.779093	1.224409	0.006963
C	-2.155816	1.205179	-0.042488
C	-2.862458	0.000020	-0.052214
C	-0.779123	-1.224422	0.006958
C	-2.155847	-1.205157	-0.042493
H	0.724969	-0.000032	1.341270
H	4.430808	-0.000651	0.965382
H	4.465057	-0.881599	-0.570948
H	4.464945	0.882337	-0.569779
H	-0.233769	2.160299	-0.000515
H	-2.693883	2.145554	-0.093109
H	-3.944765	0.000034	-0.092571
H	-0.233823	-2.160326	-0.000521
H	-2.693940	-2.145517	-0.093113

Vibrational Frequencies

1664.0796*i*	53.3853	78.9631
83.9672	175.8564	297.2018
348.4484	408.6086	438.5438
509.7140	565.4357	629.4147
662.8196	673.5704	689.2997
762.3868	827.9324	833.0764
897.3225	979.5791	984.3578
994.3667	1023.0514	1044.6337
1055.7862	1064.2582	1108.6151
1176.8571	1217.0974	1291.7240
1296.7344	1350.4755	1415.7920
1475.5283	1480.1556	1480.4291
1509.6784	1583.2755	1653.9741
2226.5675	3032.7794	3101.2147
3124.9053	3191.9425	3197.2504
3215.6683	3219.1510	3226.4217

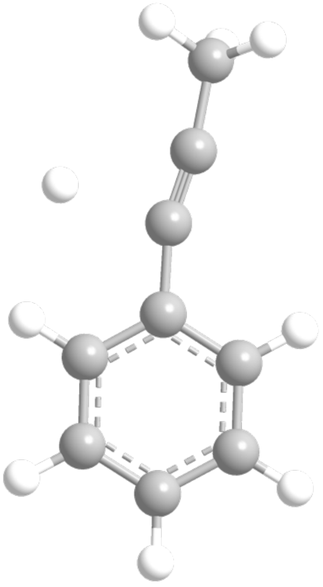

ts3, C_1, ^2A

Cartesian Coordinates

C	-4.007909	-0.154183	0.071997
C	-2.559087	-0.055222	0.035004
C	-1.371975	0.201079	-0.052189
C	0.062420	0.105366	-0.033860
H	-4.370938	-0.933037	-0.604922
H	-4.365929	-0.388546	1.079069
H	-4.460805	0.795806	-0.233466
H	-1.652295	2.040750	-0.732484
C	0.661877	-1.166905	-0.056502
C	2.044907	-1.295715	-0.020720
C	2.855466	-0.162758	0.039338
C	0.887770	1.238412	0.024873
C	2.271107	1.101902	0.062455
H	0.030416	-2.045891	-0.102366
H	2.491407	-2.283554	-0.040453
H	3.934311	-0.265529	0.066694
H	0.431660	2.220525	0.037156
H	2.894711	1.987622	0.108393

Vibrational Frequencies

589.1680*i*	34.8491	82.1934
107.6317	126.3582	240.8489
313.5079	394.0508	412.1358
418.9998	512.0155	550.2682
576.4339	637.9448	705.6860
721.6811	774.5376	853.7428
934.6307	981.3606	988.1288
1002.7227	1016.2774	1041.8484
1051.9913	1053.0975	1101.1583
1182.3546	1200.2795	1275.5598
1314.0195	1349.7078	1411.5717
1470.4686	1473.2504	1476.2821
1522.4407	1610.8188	1641.6636
2248.6079	3013.0810	3068.4667
3078.8251	3163.3861	3172.2360
3183.4852	3191.0219	3195.1591

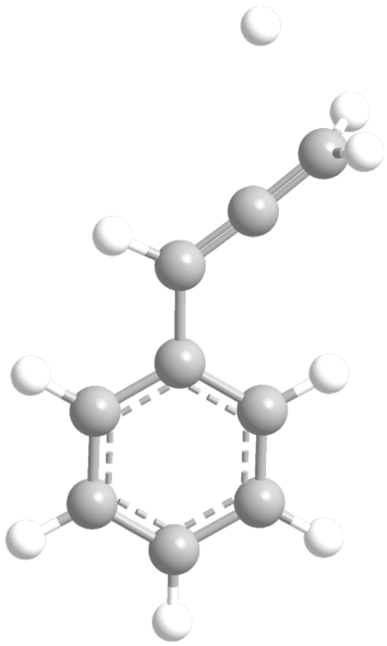

ts4, C_s, $^2A'$

Cartesian Coordinates

H	1.658586	1.786327	0.000000
C	1.430498	0.720535	0.000000
C	3.496918	-0.887421	0.000000
H	5.172550	0.470417	0.000000
C	2.443443	-0.108067	0.000000
C	0.000000	0.364342	0.000000
H	3.913582	-1.273924	0.925800
H	3.913582	-1.273924	-0.925800
C	-0.440227	-0.968320	0.000000
C	-1.796764	-1.265029	0.000000
C	-2.744198	-0.239719	0.000000
C	-0.960257	1.384982	0.000000
C	-2.320092	1.086211	0.000000
H	0.290489	-1.769544	0.000000
H	-2.119582	-2.300298	0.000000
H	-3.802386	-0.474920	0.000000
H	-0.635377	2.420364	0.000000
H	-3.047376	1.890414	0.000000

Vibrational Frequencies

361.5263*i*	55.2080	107.3461
147.8524	229.8374	231.7222
340.5541	355.5653	415.1233
448.9391	468.2793	634.8640
644.1754	649.2544	710.3120
784.3669	826.4430	853.1773
895.5091	902.7235	932.0964
980.0684	1002.2260	1015.6177
1017.3728	1048.3010	1084.2539
1109.4420	1182.1722	1201.0877
1217.4705	1310.4572	1352.4922
1375.4667	1461.3119	1492.0949
1528.6212	1621.3319	1643.5460
2002.0539	3103.9203	3108.5077
3157.3641	3163.0449	3172.7674
3180.1926	3180.2295	3190.1842

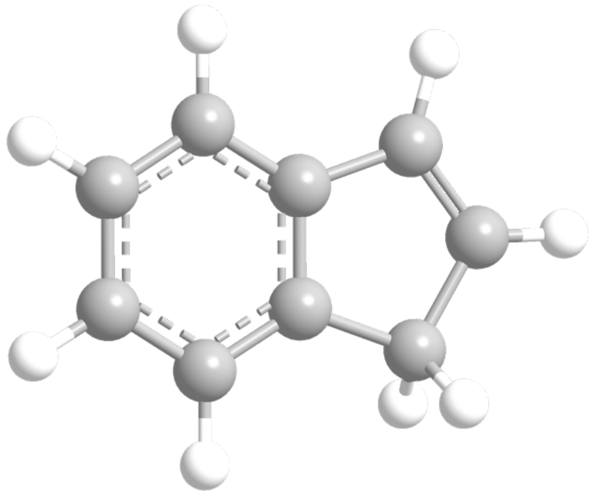

p1, C_s, $^1A'$

Cartesian Coordinates

C	2.600322	0.000025	-0.001183
C	1.393911	0.000018	-0.001004
C	4.056917	-0.000007	0.000761
C	-0.034215	0.000010	-0.000590
C	-0.751145	1.209212	-0.000114
C	-2.141228	1.205285	0.000475
C	-2.841765	-0.000015	0.000629
C	-0.751124	-1.209205	-0.000364
C	-2.141208	-1.205301	0.000218
H	4.450420	-0.018905	1.021905
H	4.451706	-0.874533	-0.524739
H	4.451651	0.893335	-0.492068
H	-0.205512	2.145160	-0.000049
H	-2.679892	2.146336	0.000895
H	-0.205470	-2.145141	-0.000502
H	-2.679856	-2.146361	0.000429
H	-3.925833	-0.000023	0.001169

Vibrational Frequencies

21.2097	93.1762	93.7504
244.9717	299.6738	396.0110
404.5500	411.6173	540.1842
549.0813	638.3122	706.8704
715.8709	774.8130	853.0617
930.9344	979.6544	986.5037
1000.9448	1016.2283	1050.0534
1051.9603	1054.2394	1100.0330
1182.2668	1200.1720	1292.2057
1309.4715	1351.2687	1416.3579
1473.3101	1478.3766	1479.2778
1525.7603	1610.0126	1643.0076
2338.0966	3019.8918	3074.1675
3079.7515	3162.5669	3171.0527
3182.7006	3190.1161	3194.7017

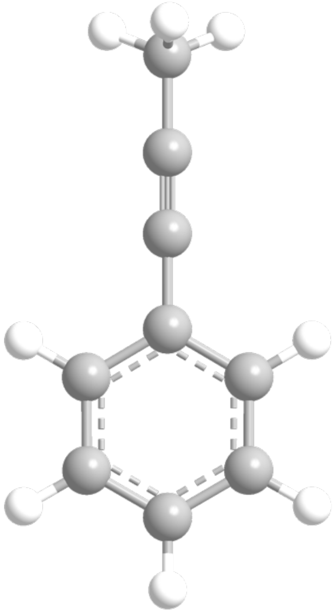

p2, C_s, $^1A'$

Cartesian Coordinates

C	0.428451	-0.617267	0.000000
C	0.000000	0.725770	0.000000
C	-0.501155	-1.655555	0.000000
C	1.893788	-0.628891	0.000000
C	2.358314	0.632232	0.000000
C	1.216877	1.620714	0.000000
C	-1.860037	-1.338688	0.000000
C	-2.282522	-0.008741	0.000000
C	-1.350923	1.034599	0.000000
H	-0.178413	-2.691147	0.000000
H	2.496275	-1.528580	0.000000
H	3.399177	0.927692	0.000000
H	1.246867	2.278669	0.877872
H	1.246867	2.278669	-0.877872
H	-2.596627	-2.134465	0.000000
H	-3.342726	0.218012	0.000000
H	-1.688191	2.066115	0.000000

Vibrational Frequencies

193.7537	211.1513	388.0297
396.5234	427.1579	542.6231
563.2046	605.1407	707.3362
734.0126	744.9778	784.0228
844.8027	871.0683	873.5357
934.9869	954.1984	956.1294
965.0813	988.2425	1042.9181
1089.0905	1132.8889	1148.6344
1177.5121	1186.0450	1227.7377
1249.4029	1314.9832	1345.4963
1391.2775	1437.2311	1489.2461
1492.6995	1603.5538	1637.3160
1652.2007	3014.1781	3035.9821
3156.3092	3162.8467	3173.7951
3186.5582	3189.5704	3213.3063

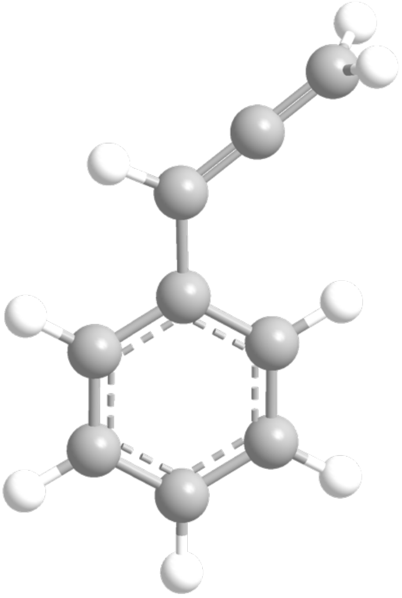

p3, C_s, $^1A'$

Cartesian Coordinates

C	-0.044382	-0.385308	-0.000002
C	-1.422262	-0.909031	-0.000001
C	-2.524717	-0.200254	0.000000
C	-3.623150	0.498088	0.000004
C	1.030177	-1.285125	0.000001
C	0.236403	0.989944	-0.000004
C	2.345303	-0.828344	0.000002
C	1.548589	1.444460	-0.000003
C	2.610582	0.538316	0.000000
H	-1.521207	-1.993331	0.000005
H	-4.103443	0.801822	0.926543
H	-4.103402	0.801898	-0.926531
H	0.830038	-2.351699	0.000003
H	-0.584049	1.698872	-0.000006
H	3.162288	-1.541344	0.000004
H	1.746826	2.510673	-0.000005
H	3.633685	0.896636	0.000001

Vibrational Frequencies

56.9901	119.6416	207.7441
324.3196	330.2122	414.1261
438.1433	450.9574	616.5055
634.8138	651.1904	710.1546
782.5122	829.6281	852.8894
880.2741	904.8768	931.6717
979.1721	1001.1483	1012.8038
1015.5828	1048.4167	1091.4951
1116.5979	1181.9685	1201.6449
1221.5889	1311.9524	1353.4123
1380.0861	1464.1815	1495.0621
1528.6797	1620.9863	1643.7970
2035.1692	3103.5321	3117.8028
3157.4002	3163.2862	3172.7226
3173.0628	3180.5393	3190.0472
